# Using a Dynamic Model to Consider Optimal Antiviral Stockpile Size in the Face of Pandemic Influenza Uncertainty

**DOI:** 10.1371/journal.pone.0067253

**Published:** 2013-06-21

**Authors:** Amy L. Greer, Dena Schanzer

**Affiliations:** 1 Modelling and Projection Section, Professional Guidelines and Public Health Practice Division, Centre for Communicable Diseases and Infection Control, Public Health Agency of Canada, Ottawa, Ontario, Canada; 2 Division of Epidemiology, Dalla Lana School of Public Health, University of Toronto, Toronto, Ontario, Canada; College of Medicine, Hallym University, Republic of Korea

## Abstract

**Background:**

The Canadian National Antiviral Stockpile (NAS) contains treatment for 17.5% of Canadians. This assumes no concurrent intervention strategies and no wastage due to non-influenza respiratory infections. A dynamic model can provide a mechanism to consider complex scenarios to support decisions regarding the optimal NAS size under uncertainty.

**Methods:**

We developed a dynamic model for pandemic influenza in Canada that is structured by age and risk to calculate the demand for antivirals to treat persons with pandemic influenza under a wide-range of scenarios that incorporated transmission dynamics, disease severity, and intervention strategies. The anticipated per capita number of acute respiratory infections due to viruses other than influenza was estimated for the full pandemic period from surveys based on criteria to identify potential respiratory infections.

**Results:**

Our results demonstrate that up to two thirds of the population could develop respiratory symptoms as a result of infection with a pandemic strain. In the case of perfect antiviral allocation, up to 39.8% of the population could request antiviral treatment. As transmission dynamics, severity and timing of the emergence of a novel influenza strain are unknown, the sensitivity analysis produced considerable variation in potential demand (median: 11%, IQR: 2–21%). If the next pandemic strain emerges in late spring or summer and a vaccine is available before the anticipated fall wave, the median prediction was reduced to 6% and IQR to 0.7–14%. Under the strategy of offering empirical treatment to all patients with influenza like symptoms who present for care, demand could increase to between 65 and 144%.

**Conclusions:**

The demand for antivirals during a pandemic is uncertain. Unless an accurate, timely and cost-effective test is available to identify influenza cases, demand for antivirals from persons infected with other respiratory viruses will be substantial and have a significant impact on the NAS.

## Introduction

Influenza has a long history in human populations. The 1918 influenza A/H1N1 pandemic resulted in millions of deaths worldwide, overwhelmed the existing health services infrastructure and resulted in significant economic losses [1]. Pandemics present an economic burden to affected countries as well as increased morbidity and mortality attributable to the emergence and subsequent global spread of a novel influenza virus. Pandemic plans outline the ways in which groups will prepare for and respond to an influenza pandemic when it occurs [2]–[4]. In order to prepare for such an event, plans need to be developed based on an estimate of the potential national impact of a pandemic. There exist a variety of freeware programs that have been used to aid planners in making these projections (e.g. FluSurge) [5]. Before the occurrence of the 2009 influenza A/H1N1 pandemic, Canada used a number of static planning assumptions resulting in plans that were based on an anticipated clinical attack rate of 15–35% [6]. Using these planning assumptions as the foundation, the Canadian Pandemic Preparedness Plan (CPIP) for the Health Sector identifies responses that may be employed during a pandemic.

The CPIP includes recommendations and guidelines for public health interventions such as vaccines and antiviral drugs [6]. Antiviral strategies are of particular importance as antivirals will be the only pharmaceutical intervention that will not require significant lead time such as the time required to produce a vaccine. The Canadian government maintains a National Antiviral Stockpile (NAS) in case of an influenza pandemic. Drugs are for the direct care of infected patients and not prophylaxis [6]. Acquiring and maintaining a stockpile for a pandemic that will occur at some unknown time in the future comes with significant challenges and costs. These include the expiry of stockpiled drugs, and the costs associated with long-term storage. A large stockpile could be very costly but a small stockpile could be insufficient if the next pandemic is very severe (e.g. with a high clinical attack rate and/or a high case fatality rate), or if a reliable, timely and cost effective point of care test for the pandemic strain is not available.

At the time of developing the 2006 CPIP, point of care tests were very promising and many jurisdictions planned to use point of care tests to screen patients for influenza during a pandemic. The decision to stockpile enough antivirals to treat 17.5% of the Canadian population was based on expert opinion after consideration of the clinical attack rate planning assumption of 15–35% and the assumption that a point of care test would be available. The evidence used to make the decision regarding the final size of the stockpile did not incorporate any information about the co-circulation of other pathogens causing respiratory disease for which antivirals may be prescribed based on symptoms alone and in the absence of any type of point of care test. As a result, the issue of antiviral wastage has not been considered in the majority of mathematical modelling papers that address antiviral stockpiles [7]–[12] and was not mentioned in the 2006 version of the CPIP. However, empirical treatment of all respiratory infections could quickly deplete antiviral stockpiles, especially if the pandemic strain emerges during the spring and activity continues into the fall.

Pandemics have occurred at 10 to 50 year intervals and are due to the emergence of a novel influenza virus subtype [13]. In past pandemics, transmissibility has been higher than for seasonal influenza, though clinical severity of the novel strains has been quite variable in comparison to seasonal influenza. For some pandemics, the burden among the elderly has been less than for seasonal influenza as a result of pre-existing immunity from previous exposure to a similar strain. However, the disease burden for younger cohorts without previous exposure has been higher than for seasonal influenza [14]. These different viral “profiles” have important implications for pandemic planning. The WHO is now encouraging countries to plan for a scalable response. In response, we have used a dynamic model to demonstrate the impact of the viral profile and concurrent interventions on the clinical attack rate, disease burden and demand for antiviral treatment. By incorporating age-specific effects we captured the impact of varying the birth cohorts with pre-existing immunity and the demand for adult or child dosing.

In this paper, we use a modified version of a previously published, age-structured, dynamic influenza model that describes the transmission of “novel” pandemic influenza viruses with different characteristics within the Canadian population combined with estimates of antiviral wastage as a result of empirical treatment of cases with influenza-like-illnesses (ILI) due to non-influenza pathogens to identify the projected national demand for antivirals in each scenario.

## Results

### Cases of Pandemic Influenza

The total number of scenarios run was 480 (5 transmission rates × 3 levels of pre-existing immunity × 2 levels of treatment seeking behaviour × 2 age-specific vaccine coverage levels × 2 wave scenarios × 4 levels of vaccine availability). In the absence of any interventions (vaccine or antiviral), the median of these 480 model generated clinical attack rates for a spring emergence and a subsequent fall wave was 36% (IQR = 28–41%; min = 22%, max = 48%). This median clinical attack rate corresponded to a scenario with a reproductive number of 1.6 and 20% pre-existing immunity in older individuals. For a fall or winter emergence, the model generated median clinical attack rate was 32% (IQR = 27–36%; min = 22%, max = 39%) over a single wave (with the median corresponding to a reproductive number of 1.6 and 40% pre-existing immunity in older individuals).

### Stockpile Size

**Figure 1:**
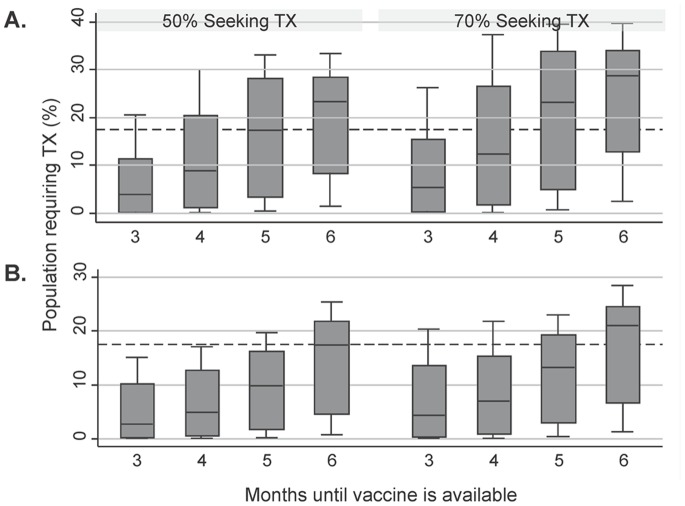
Projected range of treatment required for different pandemic wave patterns. The median (line within the shaded box), 25^th^ and 75^th^ percentile values (top and bottom of shaded box), and upper and lower adjacent values (error bars) proportion of the Canadian population expected to require antiviral treatment (Y-axis) in the presence of a safe and effective pandemic vaccine that becomes available at different points in time (X-axis). We assumed that the proportion of clinical cases seeking medical attention for their illness was 50% (left) or 70% (right). The dashed line represents the proportion of the Canadian population who would be able to be treated by our existing stockpile (17.5%). A – Fall/Winter emergence, 1 wave; B – Spring emergence, 2 waves.

Regardless of the season of viral emergence, as the proportion of clinical cases requiring care increases and vaccine availability is delayed, the proportion of the population requiring antiviral treatment increases (Figure 1). For scenarios where the virus emerges in the fall, the model projects that antiviral need in a perfect allocation scenario could be significantly higher than the current 17.5% stockpile for most scenarios (Figure 1A). In contrast, for the set of scenarios where viral emergence occurs in the spring, the decline in transmission over the course of the summer buys time to produce a vaccine, reduces the clinical attack rate and hence reduces antiviral need. For a spring emergence, a shorter time to vaccine (3–4 months) was sufficient to reduce the antiviral need in a perfect allocation scenario to below 17.5% for most scenarios (Figure 1B).

**Figure 2:**
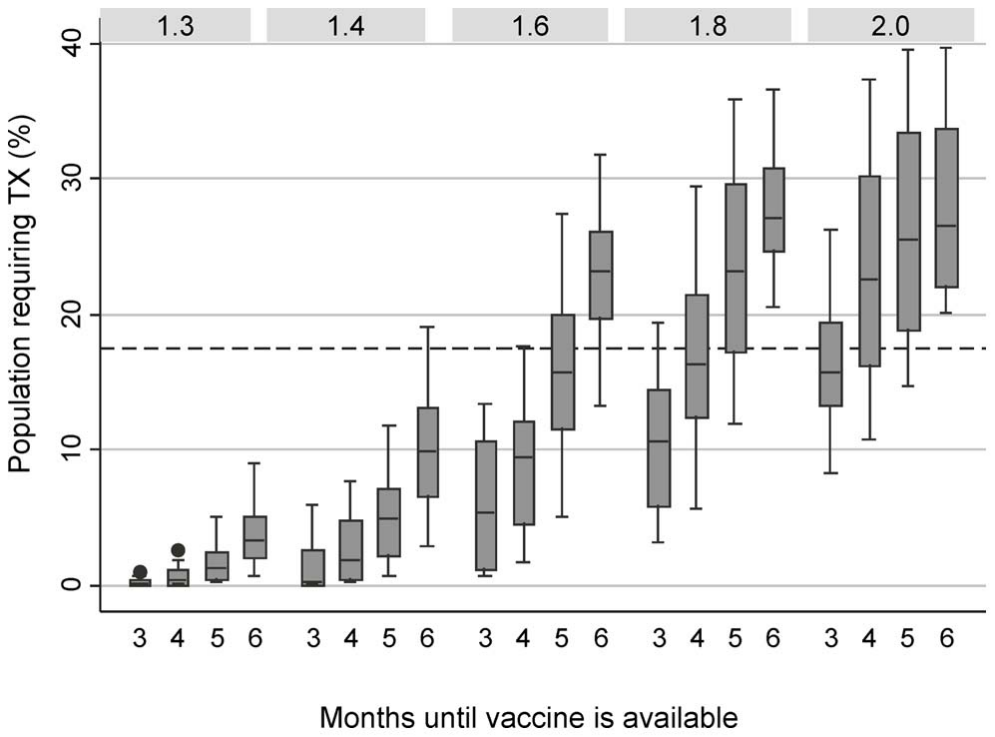
Projected range of treatment required depending on the transmissibility of the virus. The proportion of the Canadian population expected to require antiviral treatment for different combinations of model scenarios in the presence of a safe and effective pandemic vaccine when the reproductive number of the virus ranges from 1.3 to 2.0 [1.4 for seasonal influenza, 1.6–2.0 historical pandemic range]. The dashed line represents the proportion of the Canadian population who would be able to be treated by our existing stockpile (17.5%).

Combining the results for the 2 different wave patterns (Figure 1) and stratifying them by the transmissibility characteristics of the virus (R0) demonstrates that the existing stockpile size (17.5%) is sufficient for most scenarios if vaccine can be ready 3 months after viral emergence (Figure 2). However, in scenarios where vaccine is delayed (>3months), high transmissibility values (R0>1.6) result in an antiviral need that exceeds 17.5% (Figure 2). Vaccine has a more substantial impact on reducing the final size of the outbreak and therefore reducing antiviral need when R0 is low even when vaccine is available relatively late in the epidemic. At higher values of R0, the same amount of vaccine effort results in very little change to antiviral needs (Figure 2).

**Figure 3:**
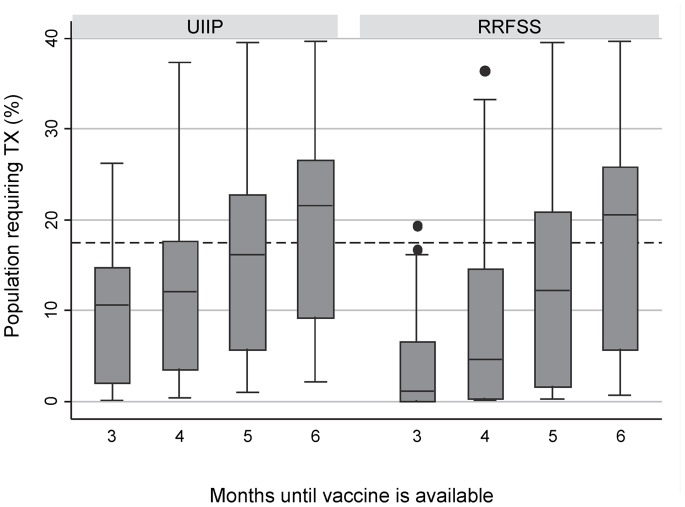
Projected range of treatment required depending on the level of vaccine coverage in the population. The proportion of the Canadian population expected to require antiviral treatment in the presence of a safe and effective pandemic vaccine when different vaccine coverage levels are considered [UIIP – Ontario Universal Influenza Immunization Program age-specific coverage, RRFSS – Ontario Rapid Risk Factor Surveillance System age-specific coverage estimates]. The dashed line represents the proportion of the Canadian population who would be able to be treated by our existing stockpile (17.5%).

The impact of increasing vaccine coverage levels is more substantial when vaccine becomes available less than 5 months after emergence (Figure 3). At 3 months post emergence, vaccine coverage at the highest level (RRFSS) drops all but one of the scenario results below the 17.5% threshold compared to coverage at UIIP levels where the upper bound remains well above 20% antiviral need (Figure 3). Moving to higher levels of vaccine coverage when vaccine becomes available >5 months post emergence, does not have a significant impact on antiviral need in the population (Figure 3).

### Baseline Respiratory Infections

In Canada, baseline (non-influenza) ILI consultation rates reach a nadir near the end of July and peak around the last week of December/first week of January with a maximum 3.4 times the minimum. Hence the number of respiratory infections occurring over the summer months is non-negligible. As a result of year round activity, the 3 respiratory infections per person per year is equivalent to 1.65 infections per person from May to December (for a spring emergence of the pandemic strain continuing with a full fall wave) and 1.48 per person for a single wave emerging in the fall or winter and a 4 month pandemic alert period. Use of the CDC definition of ILI, which includes a temperature higher than 37.8°C (100°F) plus either cough or sore throat, results in an estimate of the per capita number of self-reported ILI of 0.68 and 0.5 for the same periods. Disease severity plays a strong role in baseline rates, as illustrated in Table 1.

**Table 1:** Additional Assumptions for the calculation of antiviral stockpile: Number of baseline respiratory infections per pandemic period per capita meeting various ILI definitions.

Study	% per capita 1 wave: 4 month pandemic period (Fall/Winter)	% per capita 2 waves: Spring/Fall 9 months (May-Dec)	References	Notes
Any acute respiratory infection	148%	165%	[55], [61]	Tecumseh, Michigan study. The Fall/Winter wave includes an estimated clinical attack rate of 27% from the Tecumseh study for seasonal influenza.
Harris-Decima	95%	130%	Marek Smieja (personal communication)	Harris- Decima study definition of ILI: fever with one or more of the following symptoms: cough, headache, sore muscles, runny nose, or sore throat
CDC, Behavioral Risk Factor Surveillance System	50%	68%	[21]	CDC definition of ILI: temperature higher than 37.8°C (100°F) plus either cough or sore throat
Absenteeism due to 'flu'	30% (workforce only:12%)	41% (workforce only:16% )	[58]	Workplace absenteeism in Canada due to ‘flu’ by self report for the month of January 2010, when influenza activity was minimal. This estimate was obtained from a special question added to the Labour Force Survey (LFS), Statistics Canada. A factor of 2.5, derived from the CDC estimates of self reported ILI, was used to include similar levels of illness in children. Workplace absenteeism due to the ‘flu’ was a self assessment that the illness and absence were due to ‘flu’.

### Wastage Due to Non-Influenza Respiratory Infections

Calculating the number of antiviral treatment courses required to treat all cases of suspected viral respiratory infections (not just pandemic influenza) who present for care during the pandemic period, demonstrates the significant impact that wastage considerations can have on overall antiviral requirements (Table 2). Wastage increases the anticipated demand for antivirals during the pandemic period to a range of 65% to 144% of the Canadian population compared to 0.1% to 40% as was the case with the assumption of perfect allocation (Table 2).

**Table 2:** The median, minimum and maximum of the proportion of the Canadian population (%) that may require antiviral treatment during a pandemic after considering the circulation of non-influenza viruses that produce acute respiratory symptoms (including any of the following: runny nose, sneezing, sore throat, cough, cervical lymphadenopathy, fever, malaise, myalgia, loss of appetite, headache, and/or chills for scenarios where viral emergence occurs in the spring or in the fall/winter [61].

SPRING EMERGENCE		Min (3 m)	Median (3 m)	Max (3 m)	Min (6 m)		Median (6 m)	Max (6 m)
50% of cases with acute respiratory symptoms seek medical attention	Perfect allocation (TX only pandemic cases)	0.1	2.8	15.0	0.8		17.4	25.4
	Imperfect allocation (TX all cases).	82.6	85.3	97.5	83.3		99.9	107.9
70% of cases with acute respiratory symptoms seek medical attention	Perfect allocation (TX only pandemic cases)	0.1	4.4	20.3	1.3		21.1	28.5
	Imperfect allocation (TX all cases).	115.6	119.9	135.8	116.8		136.6	144.0
**FALL/WINTER EMERGENCE**		**Min (3 m)**	**Median (3 m)**	**Max (3 m)**	**Min (6 m)**		**Median (6 m)**	**Max (6 m)**
50% of cases with acute respiratory symptoms seek medical attention	Perfect allocation (TX only pandemic cases)	0.03	3.9	20.5	1.5		23.3	33.4
	Imperfect allocation (TX all cases).	65.0	68.9	85.5		66.5	88.3	98.4
70% of cases with acute respiratory symptoms seek medical attention	Perfect allocation (TX only pandemic cases)	0.1	5.4	26.3		2.5	28.7	39.8
	Imperfect allocation (TX all cases).	91.1	96.4	117.3		93.5	119.7	130.8

TX = treatment, m = months until vaccine availability in Canada.

## Discussion

Using a dynamic influenza disease transmission model structured by age and chronic health conditions we have described the transmission of “novel” pandemic influenza viruses with different characteristics within the Canadian population. We have used the model to identify the size of the NAS required to meet the national antiviral need in each scenario in circumstances where we have both perfect and imperfect allocation of antivirals to true pandemic influenza cases. Previous modelling work to examine optimal allocation of an antiviral stockpile within a population has not included the possibility of patients presenting for medical care who are infected with a respiratory pathogen that is not pandemic influenza [9], [15]–[19]. By leaving out this important biological component of the population disease dynamics during a pandemic the projected size of antiviral stockpile required is underestimated. Our findings provide important insight into the impact of imperfect allocation of antiviral treatment.

### Effect of Virus Characteristics

The transmissibility characteristics of a novel pandemic influenza strain upon emergence are impossible to predict. However, historical pandemics have yielded a range of plausible values for the transmissibility of pandemic strains that have been encountered to date. Clearly the characteristics of the virus as well as the level of pre-existing immunity that exists in the population will strongly influence both the final size of the epidemic and the time over which the epidemic occurs. In instances where transmissibility is high (R0 = 2.0) and there is no pre-existing immunity, the virus spreads rapidly throughout the population. In contrast, a low R0 combined with significant pre-existing immunity can result in an epidemic that climbs more slowly and is more prolonged. The implications of these differences in virus characteristics are seen in the way that the characteristics may interact with intervention strategies such as antiviral treatment and vaccination. In addition, differences in the timing of the pandemic waves as a result of a reduction in transmission rates over the summer resulted in waves that were consistent with the timing of peak activity in Canada in 2009. In contrast, when the novel strain emerges during the fall or winter season, a single large wave is expected, though some jurisdictions have observed multiple waves. These different time scales can significantly affect the observed impact of an intervention strategy such as vaccination because the success of the intervention is determined in part, by how much of the population has already been naturally infected at the time the intervention begins.

### Effect of Vaccination

For both the spring and fall/winter emergence scenarios, the impact of vaccine is most significant when vaccine becomes available as early as possible. In scenarios where vaccine becomes available 6 months after viral identification, the impact of vaccine on antiviral need is very modest, as most scenarios have the pandemic peaking within 6 months of detection of imported cases. Current advances in vaccine technology that attempt to move from egg-based, vaccine production to cell-based, or plant-based, vaccine production show great promise and could likely produce vaccine within this shorter timeframe [29]–[30], increasing the odds that a vaccination program could be rolled out before the natural pandemic peak. Sensitivity analysis to examine the impact of vaccine efficacy (VE) demonstrated that even if VE were higher than 70% [20], the impact is largely overshadowed by the long period required to begin the vaccine program if vaccine takes 6 months to produce. Improved VE combined with earlier vaccine availability decreases the need for antivirals even more than what is reported here.

### Wastage Considerations

To date, the size of the National Antiviral Stockpile (NAS) has focused on the number of antiviral courses that would be required to treat symptomatic cases of pandemic influenza in Canada [7]. However, there are other pathogens which also produce similar respiratory symptoms. Wastage under a strategy that attempts to treat all patients with respiratory symptoms who might present for treatment results in antiviral need that in some circumstances could exceed 100% of the Canadian population. Using a more specific case definition such as that used by the United States Centre for Disease Control (CDC) (temperature higher than 37.8°C (100°F) plus either cough or sore throat) [21] should reduce wastage, however, this also means that some proportion of true pandemic influenza cases would also present missed treatment opportunities due to a lack of fever (especially in older adults) [31]. However, one study found that only 58% of campers who tested positive for the 2009 pandemic strain had a fever, [22] and ILI criteria are not always met in hospitalized patients who test positive. Despite, interest in using point of care testing during a pandemic, a previously published decision analytic model has demonstrated that using near-patient testing as a triage method for managing a stockpile of antiviral drugs is unlikely to be a cost-effective mechanism for conserving drugs [23]. In fact, Siddiqui and Edmunds (2008) have demonstrated that it is more cost-effective to increase the size of the stockpile in case of a higher clinical attack rate than to rely on near-patient testing [23]. A strategy that includes a more strict case definition could result in increased morbidity and mortality [24]–[28].

Our results do demonstrate that stockpiling for a “treat all” approach may not be logistically feasible and therefore, alternatives may need to be considered such as targeting specific sub-groups or examining creative procurement arrangements to reduce upfront stockpile requirements. In any case, ethics must be considered. Further research to identify clinical symptoms that would help to target individuals who would benefit the greatest from antiviral treatment would help reduce wastage.

### Limitations

We have not included any possible impacts of non-pharmaceutical public health measures. Non-pharmaceutical intervention measures range from public health messaging (e.g. hand hygiene and cough etiquette) to requests to stay home when ill, school closure, isolation and quarantine. Ultimately, it is difficult to predict the way that individual Canadians might behave in a pandemic and behaviour will be driven by each individual’s perception of risk over the course of a pandemic. For this reason, we have not included non-pharmaceutical measures in this model.

We have also not explicitly considered the possible impact of antiviral resistance. Stockpiles will likely be comprised of several different antiviral drugs (e.g. amantadine, oseltamivir, and zanamivir) as a way to hedge against the possibility of a resistant strain emerging or that the new strain develops resistance as a result of aggressive antiviral use in the community. Clearly, decisions about stockpile size and composition cannot be made without considering the risk of antiviral resistance.

All models require simplifications and assumptions and we have taken steps to root our simplifications and assumptions in the best available evidence. In the case where evidence was lacking, we consulted clinical experts in order to make choices that were realistic however, important uncertainties remain for specific assumptions that are important to highlight. All of our vaccine timing scenarios assume that the strain emerges in Canada shortly after it is first identified as a strain with pandemic potential. It seems likely that if the virus were to emerge outside of Canada (e.g. southeast Asia), there may be more lead time before the novel strain was introduced to Canada than we saw in 2009. In this case, vaccine development would already be underway. If this were the case, Canada would receive vaccine at an earlier stage of the epidemic. Our findings make the case for pursuing technological advances in vaccine production and distribution. Vaccination remains the cornerstone of public health and as such, improving our ability to offer vaccine early in an epidemic as well as having antiviral stockpiles to treat symptomatic individuals in order to prevent them from serious morbidity and mortality will have significant positive effects on the health of all Canadians when the next pandemic occurs.

### Conclusions

This work demonstrates the utility of incorporating transmission dynamics, disease severity, bundled intervention strategies, and wastage into pandemic planning discussions. Since the characteristics of the next pandemic influenza strain will be unknown until the virus emerges, this dynamic methodology allows decision-makers to examine wide-ranging scenarios in order to make informed decisions in the face of uncertainty. The demand for antivirals during a future pandemic is uncertain. In the absence of an accurate, timely and cost-effective point of care test to identify influenza cases, demand for antivirals from persons infected with other respiratory viruses will be substantial. Once new technology reduces the production time of influenza vaccines from 6 to 3 months, antivirals will still be needed for the first wave, and in temperate climates, the first wave may be a full pandemic wave if the virus emerges during the fall or winter. Further research in areas such as improving the sensitivity of point-of-care laboratory tests for influenza and into identifying functional status indicators at time of symptom onset that predict severe disease outcomes could reduce the potential demand for antivirals stockpiles.

## Methods

### Model Structure

We developed a deterministic, SEIR compartmental model based on our previously published model for pandemic influenza [29]. The model assumed that all Canadians were in one of several, mutually exclusive health states at any given point in time. Initially, individuals without pre-existing immunity are considered susceptible to infection (*S*), while those with pre-existing immunity are placed in the recovered compartment (*R*). Once exposed, individuals move from the *S* to the *E* compartment, until they become infectious *(I)*. The previously published model [29] was modified to include three different “Infected” compartments. Individuals could be asymptomatically infected (*I_A_*), symptomatically infected but never treated with antivirals (*I_S_*), or symptomatically infected and treated with antivirals (*I_T_*). Lastly, recovered individuals moved to the *R* compartment. Re-infection of previously infected individuals was not included in the model. We assumed that 40% of all infected individuals were asymptomatic but that there was no differential transmissibility between the two groups [30]–[31]. Population data was from the 2006 Canadian Census [32]. The timing of the initial cases was based on data for imported cases in Canada during the 2009 pandemic [29]. The model ran for 12 months following the initial introduction of the pandemic influenza strain to Canada.

### Age Structure

The model was age-structured (0–4, 5–13, 14–17, 18–23, 24–52, 53–64, 65+) and age-specific mixing patterns were based on empirical data from Mossong et al. [33]. To account for individuals with chronic, underlying medical conditions, the proportion of each age group with at least one chronic condition for which seasonal influenza immunization is recommended (asthma, emphysema, chronic obstructive pulmonary disease, diabetes, heart disease, cancer, and stroke) was estimated from the Canadian Community Health Survey (CCHS) [34]. The elevated risk to pregnant women in the second and third trimester was accounted for by a separate health state for pregnancy (*P*). The population estimated to be in this state at any given point in time was derived from Canadian census data for pregnancies and live births [35]–[36]. Health state and pregnancy categories were used to set priorities for interventions.

### Pre-existing Immunity

In the model, we varied the proportion of individuals aged 65 and older who were not susceptible to infection by the circulating pandemic strain as a result of previous exposure to a similar influenza strain from 0–40%. Since identifying individuals with pre-existing immunity is not possible, intervention strategies were applied equally based only on health status and age.

### Influenza Transmissibility, Natural History and Clinical Characteristics

We examined the impact of using a range of basic reproductive numbers (R0) from 1.3 to 2.0, derived from the epidemic growth rate of historic pandemics [9], [30], [37]–[44], and included scenarios with transmission rates similar to seasonal influenza [45]. All natural history parameters and ranges examined in the model are outlined in Table 3.

**Table 3:** Parameter values and assumptions used for the Canadian antiviral stockpile model.

Item	Strain	Value	Reference(s)
**TRANSMISSIBILITY**			
R0	2009	1.3	[37]
	Seasonal	1.4	[45]
	1957/1958	1.6	[9], [30], [39]–[40], [42]
	1968/1969	1.8	[30], [39]–[40], [42]–[44]
	1918	2.0	[30], [38]–[42]
**NATURAL HISTORY**			
Latent period	Seasonal	2.1 days	[45]
Duration of infection	Seasonal	4.8 days	[45]
Pre-existing immunity in individuals >65 years		0% (0–40%)	Assumption
**CLINICAL CHARACTERISTICS**			
Proportion symptomatic	1957	60%	[30]
Proportion of symptomatic cases seeking medical attention	1957	50% (50–70%)	[30]
**VACCINATION**			
Vaccine coverage by age group	Age group	UIIP (%) [59]–[60]	RRFSS (%)*
	0–4	26	60
	5–13	30	60
	14–17	31	60
	18–22	29	62
	23–52	29	54
	53–64	47	65
	65+	75	75

UIIP – Universal Influenza Immunization Program.

RRFSS – Rapid Risk Factor Surveillance System.

* RRFSS Module – Ontario Ministry of Health and Long Term Care and Public Health Ontario.

### Seasonality

Significant uncertainty exists regarding where and when a novel pandemic influenza strain may emerge or be introduced. Influenza epidemics typically peak during January or February in Canada, though recent data have shown peaks as early as November, and as late as April in some communities [46]. The reasons for the observed seasonality of influenza in temperate climates is poorly understood, though may be due to reduced transmission rates over the summer, changes in environmental factors (e.g. humidity) or contact patterns (e.g. school holidays) [47]–[51]. To force seasonality to align with the spring/fall wave phenomenon observed in the northern hemisphere in previous pandemics, transmissibility was decreased from July to early September. We included two scenarios: a spring/fall scenario with two waves; and a fall/winter scenario with one wave in either the late fall or winter.

### Vaccination

All scenarios evaluated included some form of vaccination. We assume that the pathogen would be identified as the first cases are imported to Canada and the vaccine would be available for distribution within either 3 months or 6 months of the first imported cases. Currently available technology is egg based, and vaccine production took approximately 6 months in 2009. Non-egg based technologies are under development and would likely require a three month production and approval period [52]–[53]. We assumed that it will take an additional 6 weeks to fully roll out a pandemic vaccination program. Vaccine efficacy in the model was set to 70% and the time required to develop full immunity post vaccination was assumed to be 10 days [29]. Vaccine prioritization was defined according to the 2009 experience in Canada: 1) pregnant women and all individuals with a chronic underlying condition as defined by the CCHS (regardless of age), 2) healthy children aged 0–4 and healthy adults aged 65+, 3) healthy children aged 5–17, and 4) healthy adults aged 18–64. Vaccine coverage levels by age group were set to levels for the 2009 pandemic [29] (Table 1).

### Antivirals

Antiviral use was captured in the model by including a compartment for infectious individuals who were treated (*I_T_*). We assumed that 50–70% of symptomatic individuals would request antiviral therapy [30] (Table 1), and that for ethical reasons, all individuals with respiratory symptoms who requested treatment would be eligible to receive antivirals. In addition, we assumed that antiviral treatment would not decrease the risk of transmission to others. The model assumes that all treated individuals receive a 5 day course of antivirals [54]. Longer durations of treatment or higher dosages for severely ill patients were not considered.

### Antiviral Wastage

To assess treatment courses required to treat non-influenza viral infections, it was assumed that the public would be encouraged to seek antiviral treatment for respiratory infections as soon as possible after symptom onset during the pandemic period. The most definitive study on the number of respiratory infections per person per year that we identified is the Tecumseh, Michigan study [55]. The 3 respiratory infections per person per year, less 9% that were identified as infections with a seasonal influenza strain, were prorated to the pandemic period, using the seasonality of medical consultations for respiratory infections, or more specifically, influenza like illness (ILI) consultation rates per 1000 patient visits as reported to *FluWatch* [56]. The weekly seasonality of ILI infections due to viruses other than influenza was estimated from this time series using a Poisson regression model similar to the models used to estimate hospital admissions [57] and absenteeism attributable to influenza [58]. For the fall/winter scenario, seasonal influenza infections were included in the baseline estimate as a non-pandemic respiratory illness. We assumed that all persons visiting their doctor for an ILI would be equally likely to request antiviral treatment based on the severity of the symptoms and health status, rather than whether the infection was actually due to the pandemic strain. Baseline rates were estimated for a range of disease severity criteria (Table 2).
